# The wicked ocean

**DOI:** 10.1007/s13280-017-1000-0

**Published:** 2017-12-23

**Authors:** Øyvind Paasche, Erik Bonsdorff

**Affiliations:** 1 Environmental and Marine Biology, Faculty of Science and Engineering, Åbo Akademi University, Artillerigatan 6, 20520 Åbo, Finland; 2 Bjerknes Centre for Climate Research, Allégaten 70, 5020 Bergen, Norway

Anthropogenically induced climate change has created a set of intriguing scientific problems pertaining to the Seas and the Oceans on earth that can be monitored, analysed, modelled and consequently understood at some level. The international research community is deeply engaged in this endeavour as exemplified by the United Nations Decade of Ocean Science for Sustainable Development (2021–2030). Even so, the types of problems caused by human activity are inherently difficult to solve, if solvable at all, making them equally difficult to approach and manage. Here we address these as ‘wicked problems’ (first discussed by Churchman 1967, and later defined and formalized regarding social and natural sciences by Rittel and Webber 1973), posing challenges that currently seem to surpass our ability to tackle them.

We see the Sea and the Oceans, but mainly from above—we see only the surface. A curved and broken line that separates our world from the one below, from the life in and of the Sea. Seen from ashore, its beauty and incessant perplexity is staggering—an outlook which we increasingly seek refuge in, for recreation and for well-being (see for instance Bowen et al. 2014).

Our land-based perspective is rewarding, but also limiting in its lack of three-dimensionality, because the vastness of the Seas and the Oceans is so much more than the surface we observe from a distance. It is what we cannot see which makes it so challenging to understand. Water stretches across basins and beneath ice shelves, extending all the way down to the abyssal depths. The Seas and Oceans cover over 70% of Earth’s surface or 3.61 × 10^14^ km^2^ with a total mass of 1.4 × 10^21^ kg (Vallis 2011). It has a mean depth of over 3.7 km, but even so all water on Earth can still be fitted in a sphere of only 1400 km in diameter. Despite big numbers, clean water is a scarce commodity that needs to be treated accordingly.

Ocean circulation and overturning, processes that involve wind-driven gyres, turbulent diffusion and the sinking of surface water, are unevenly distributed globally. Gebbie and Huybers (2011) estimate that 15% of the total ocean’s surface accounts for 85% of the production of deep water. Despite the fact that this production is localized to a few high latitude areas, it still enables continued water mass exchange between the major interhemispheric basins.

This vital engine helps maintain a balance in earth’s climate by constantly moderating it through uptake of heat and subsequent redistribution. On a human time scale, the ocean engine is slow, given the volume in question, explaining why it can take up to 1200–1500 years before submerged surface water in the Pacific Ocean reach the deepest parts of the basin (Gebbie and Huybers 2012) or even longer before it resurfaces, all depending on which ocean and water mass you examine (Wunsch and Heimbach 2008).

It is that sluggishness we have come to rest the fundaments of our fast-growing and fast-living civilizations on because if the heat absorbed was released back from the ocean on shorter time scales the impact of our expanding footprint would be immediately evident. This is but one explanation for why we have taken the goods and services provided by the Seas and Oceans for granted through the centuries, a type of behaviour which has cemented a view that is hard to change. Numerous species have been decimated until saved in the last hour like the Antarctic blue whale (Attard et al. 2016) or the sea otter (Doroff et al. 2003). The list of threatened species is growing by the day which in effect makes the global marine ecosystem less resilient to further change (Levin and Lubchenco 2008).

The Ocean’s great storage capacity is something we have exploited to the full, and have done for quite some time. For much too long we have believed in the concept that “dilution is the solution to pollution”, and that the resources in the ocean, though plentiful, would be endless. Surplus heat and CO_2_ are absorbed and funnelled to the darkest nadirs, not to return to the surface before centuries have passed. The dire side of the industrial revolution is conveniently hidden from our naked eye—if we don’t see it, it’s easier to pretend it’s not there.

Even so, there is an increasing interest in the Oceans and the Seas, politically as well as commercially. The paradox is inescapable, encapsulating our contorted modernity. We undermine its functions at the same time as we expect ever more from it. Because more food needs to be marine, and as we harvest down the food web, we inevitably make recovery slower and more uncertain. Removing large predatory mesopelagic fish (i.e. top predators) will alter the entire marine food web through trophic cascades, including pelagic primary production, and might have unforeseen impacts on the general energy flow of the entire marine ecosystem (van Denderen et al. 2017) and on their role in capturing and exporting CO_2_ from the surface (Pauly et al. 1998; Daskalov et al. 2007). Goods must be transported with ships (already 90% of global trade is by sea (Valentine et al. 2013)), and atmospheric CO_2_ must be taken up, preferentially at the same pace as now or faster. We have created a series of wicked problems, where the solutions we propose oftentimes trigger new problems, or are merely covering up for old sins.

Despite the fact that sea level since 1993 has risen with 3.1 mm ± 1.4 mm y^−1^ (Dangendorf et al. 2017) enhancing the risk of costal hazards, the coastal population of the planet is on the rise. The fraction of humans now living less than 100 km from the coast is at least 40% (Small and Nichollis 2003; http://www.un.org/esa/sustdev/natlinfo/indicators/methodology_sheets/oceans_seas_coasts/pop_coastal_areas.pdf), and scenarios suggest that the trend is steepening (Neumann et al. 2015).

Our systematic exploitations have had, and continue to have severe effects on marine and terrestrial ecosystems and on global climate itself. Some of these effects are secondary, and we have barely seen the manifestation of them yet. Many of these problems are wicked: they are moving targets that cannot be fully solved on human time scales, and the success of whatever potential solutions we come up with are contingent on how we frame them and what type of conflict of interest they embed in the first place.

On a geopolitical level the UN’s 17 Sustainable Development Goals are important, embodying a moderate progress. They are written in the expectant language that international politics demands. Goal #14, curiously dubbed ‘Life *below* water’, is all about the Oceans and the Seas. Target 14.3 states that we need to « Minimize and address the impacts of ocean acidification, including through enhanced scientific cooperation at all levels. » (https://sustainabledevelopment.un.org/sdg14).

As opposed to many other consequences of industrial CO_2_ emissions, ocean acidification is easier to grasp, but harder to reverse. More CO_2_ in the atmosphere due to combustion of fossil fuels equates with more uptake of CO_2_ by the Oceans (e.g. Le Quéré et al. 2017). This gradually affects and ultimately lowers the pH value of the ocean, which in turn contributes to ocean acidification. Observations confirm this (e.g. Sabine et al. 2004). Observations also confirm that we keep on emitting CO_2_ at a surprisingly high rate (as if the currency of scientific knowledge carries limited value for political decision-making and subsequent societal action), and once the CO_2_ is absorbed by the upper layer of the ocean it cannot easily be undone.

There is no backdoor out of this wicked problem, which we barely understand the range or severity of. There is no silver bullet that will present a universal remedy for the wickedness. Having a political target that depends upon ‘enhanced scientific cooperation at all levels are’ is courteous to the science community, but will do little to ‘minimize the impact’ or, more importantly, halt the current development on human time scales unless other agents and processes put that insight to good use.

Acknowledging the problem, making it emblematic, but doing next to nothing is confusing and demotivating. It paints a contradictory picture of reality that obfuscates a clear understanding of what the crux of the problem is: our continued emissions of greenhouse gases, our continued harvests and usage of ecosystem goods and services. And by doing so, it becomes increasingly harder for the public to identify pathways to action that at least resolve parts of the problem. We need an opening that shows the way forward, and we need positive and sustainable examples based on scientific knowledge, implemented at full scale rather than just demonstration-projects. An equivalent to *The Montreal Protocol on Substances that Deplete the Ozone Layer*, only for the ocean, would serve the purpose only if it were fast enough. Precisely how *The Paris Agreement* will be played out in terms of reduced CO_2_ emissions remains to be seen, as is true for the COP23 meeting in Bonn in November 2017, but atmospheric concentrations of CO_2_ will continue to soar in decades to come and have in fact increased for 2017 as well (Le Quéré et al. 2017).

Global sea level rise is another wicked problem. There are many others including deoxygenation (Keeling et al. 2010) and plastic debris (Law 2017), which have little in common except that they need to be addressed and managed. Reaching an international agreement on marine plastic pollution could be the first important step towards a less polluted scenario (Borelle et al. 2017). As for deoxygenation, it is barely mentioned in governmental papers (Gallo et al. 2017). Other issues, like temperature and sea level, are possibly better known outside the science community.

Consider the fact that since the mid-20th century over 90% of the excess heat in the atmosphere due to greenhouse gases has been absorbed by the Oceans and during the last century or so surface waters have warmed by 0.7° ± 0.08 °C (Jewett et al. 2017). The phenomena mentioned here constitute problems that neither are easily addressed nor solved. A task further complicated by the sheer number of stakeholders involved because we all need to agree. The reality of rising sea level will not quietly go away, not even when it finally stabilizes hundreds of years from now and our sustainable targets will since long have elapsed and many coastal settlements have been abandoned. Part of the surplus heat once absorbed at the ocean surface will by default resurface and by then be someone else’s problem to handle.

The Oceans and the Seas are sometimes posed as a potential redeemer of the perilous climate and environmental challenges we are currently facing only if we treat it right (Cf. OECD 2016)—a message echoed by governments and international bodies. If that truly is the case, then we need to start addressing it with the resources and attention it necessitates. As of now, we still behave as if there is little urgency associated with the wicked problems described here, but urgency can also result in ad hoc solutions or statements with little or no positive impact.

We need serious and solid science-and-society interactions to grasp, comprehend, communicate and ultimately perhaps try to tame some of the wickedness of the anthropogenized Ocean. A step in the right direction would, according to Frame (2008), be to build “a constituency that is willing to think beyond the next generation”. Such a view arguably permits a wider perspective on several of the issues raised here and can perhaps replace the sense of disbelief that haunts the ongoing debate about the ocean and by so doing help create new pathways to action.
